# On the Use of Barium, Cadmium and Nickel in Pharmacy

**Published:** 1852-08

**Authors:** 


### CHEMISTRY.

*On the Use of Barium, Cadmium and Nickel in Pharmacy.*—Barium and its compounds, more particularly the chloride of barium (which, by the bye, has been struck out of the text of the new *Pharmacopœia Londinensis*, and merely placed among the tests in the Appendix,) is just now attracting the attention of physicians, as applicable to those diseases in which the iodides of iron and of potassium, and the tincture of sesquichloride of iron, have been administered with such decided advantage.

The salts of two other metals are likely to come into considerable use as articles of the *Materia Medica.* The metals alluded to are Cadmium and Nickel, of which the sulphates have been lately employed with advantage by some distinguished physicians, as serviceable therapeutic agents, more particularly, it is said, in some diseases peculiar to females.—*Ibid, from Annals of Pharmacy.*

